# The ubiquitin ligase Siah is a novel regulator of Zeb1 in breast cancer

**DOI:** 10.18632/oncotarget.2696

**Published:** 2014-12-18

**Authors:** Anna Chen, Christina S.F. Wong, Mira C.P. Liu, Colin M. House, Jaclyn Sceneay, David D. Bowtell, Erik W. Thompson, Andreas Möller

**Affiliations:** ^1^ Cancer Genomics and Genetics Laboratory, Peter MacCallum Cancer Centre, St. Andrews Place, East Melbourne 3002, Australia; ^2^ Department of Pathology, The University of Melbourne, Parkville 3010, Australia; ^3^ Tumour Microenvironment Laboratory, QIMR Berghofer Medical Research Institute, Herston 4006, Australia; ^4^ Sir Peter MacCallum Department of Oncology, The University of Melbourne, Parkville 3010, Australia; ^5^ Department of Biochemistry, The University of Melbourne, Parkville 3010, Australia; ^6^ The University of Melbourne Department of Surgery, St Vincent's Hospital, Fitzroy 3065, Australia; ^7^ St Vincent's Institute of Medical Research, Fitzroy 3065, Australia; ^8^ Institute of Health and Biomedical Innovation and School of Biomedical Sciences, Queensland University of Technology, Kelvin Grove 4000, Australia

## Abstract

Elucidating the mechanisms that underlie metastasis is of paramount importance to understanding tumor progression and to the development of novel therapeutics. Epithelial to Mesenchymal Transition (EMT) plays a vital role in tumor cell dissemination and is regulated by a core cassette of transcription factors. Despite recent advances, the molecular pathways that regulate the EMT program have not yet been fully delineated. We show that Siah ubiquitin ligases regulate Zeb1 protein, a key EMT transcription factor. The induction of EMT in breast cancer cells leads to the down-regulation of Siah, while the loss of Siah induces a mesenchymal phenotype, concurrent with an up-regulation of Zeb1. Overexpression of Siah *in vitro* mediates Zeb1 degradation, which can be blocked with a Siah peptide inhibitor. Thus, this work demonstrates that Siah is a novel regulator of EMT. This work is the first to identify a mechanism of post-translational regulation of the key Epithelial to Mesenchymal Transition transcription factor Zeb1.

## INTRODUCTION

Epithelial to Mesenchymal Transition (EMT), a program during which epithelial cells undergo major molecular, biochemical and morphological changes to adopt a mesenchymal phenotype, plays a role in facilitating tumor cell dissemination [1]. EMT, a highly conserved process across species, is vital during early developmental stages and is typically characterized by the loss of epithelial-specific proteins such as E-cadherin and by increased expression of mesenchymal markers [1]. In the cancer setting, by undergoing EMT, tumor cells are equipped with a migratory and invasive phenotype, thereby enhancing cell dissemination from the primary tumor, entry into the circulation, and survival in the circulation and in disseminated sites. EMT also reduces cell proliferation and inhibits senescence and apoptosis, thus conferring resistance to conventional chemotherapies [1, 2]. Triggered by a number of growth factors, most notably Transforming Growth Factor-beta (TGF-β), EMT is regulated by a core cassette of transcription factors including Zeb1, as well as Snail (Snail1), Slug (Snail2), Twist1/2 and Zeb2 [3].

Recently published work suggests that Zeb1 is the most proximal EMT transcription factor in breast cancer, acting via the repression of E-cadherin and the microRNA-200 family [4–7]. Aberrant Zeb1 expression has been described in a number of other cancers, including colorectal cancer, metastatic lung cancer and aggressive uterine cancer [8, 9]. As such, pathological EMT is currently being investigated as a candidate for therapeutic intervention in cancer patients [1, 10, 11]. While post-translational regulatory mechanisms involving ubiquitination and proteasomal degradation of Snail, Slug, Twist and Zeb2 have been described, the regulation of Zeb1 protein abundance is currently unknown [12–15].

On the other hand, Siah proteins have been previously shown to play a role in tumorigenesis and metastasis [16, 17], however, their involvement in EMT has not yet been described. As a family of E3 ubiquitin ligases, Siah proteins transfer ubiquitin onto substrates, thereby targeting them for degradation by the 26S proteasome [16]. In humans, the Siah family consists of two members, Siah1 and Siah2, whereas in mice there are three members, Siah1a, Siah1b and Siah2 [16]. Interestingly, Siah has been reported to act as both an oncogene and a tumor suppressor [17]. As an upstream regulator of the Sprouty2/Ras, hypoxia-inducible factor (HIF) pathways and of the HIPK2 tumor suppressor protein, Siah is able to modulate tumor progression [18–22]. Accordingly, some studies have shown that Siah2 blockade inhibits tumor development and metastasis [19, 23, 24]. Conversely, some reports have indicated that Siah, in particular, Siah1, may function in a tumor suppressive manner, by inducing apoptosis and sharing similar mechanisms as p53 and p21 [25, 26]. Additionally, Siah1 has been demonstrated to be involved in the degradation of β-catenin, through interaction with adenomatous polyposis coli and p53, in several cancer cell types [27–32]. While Siah does appear to have contradictory roles in cancer, its involvement in several critical signalling pathways makes it an interesting target to study in the preclinical and clinical settings.

Here, we show that Siah ubiquitin ligases regulate Zeb1 protein levels. The induction of EMT in human epithelial breast cancer cells leads to the down-regulation of Siah protein expression, while the loss of Siah in these cells and in murine breast cancer cells results in a mesenchymal phenotype. These data identify Siah as a novel regulator of EMT by controlling the abundance of the key transcription factor Zeb1, while Siah itself is subject to regulation by EMT-inducing factors.

## RESULTS

### EMT induction causes the down-regulation of Siah

**Figure 1:**
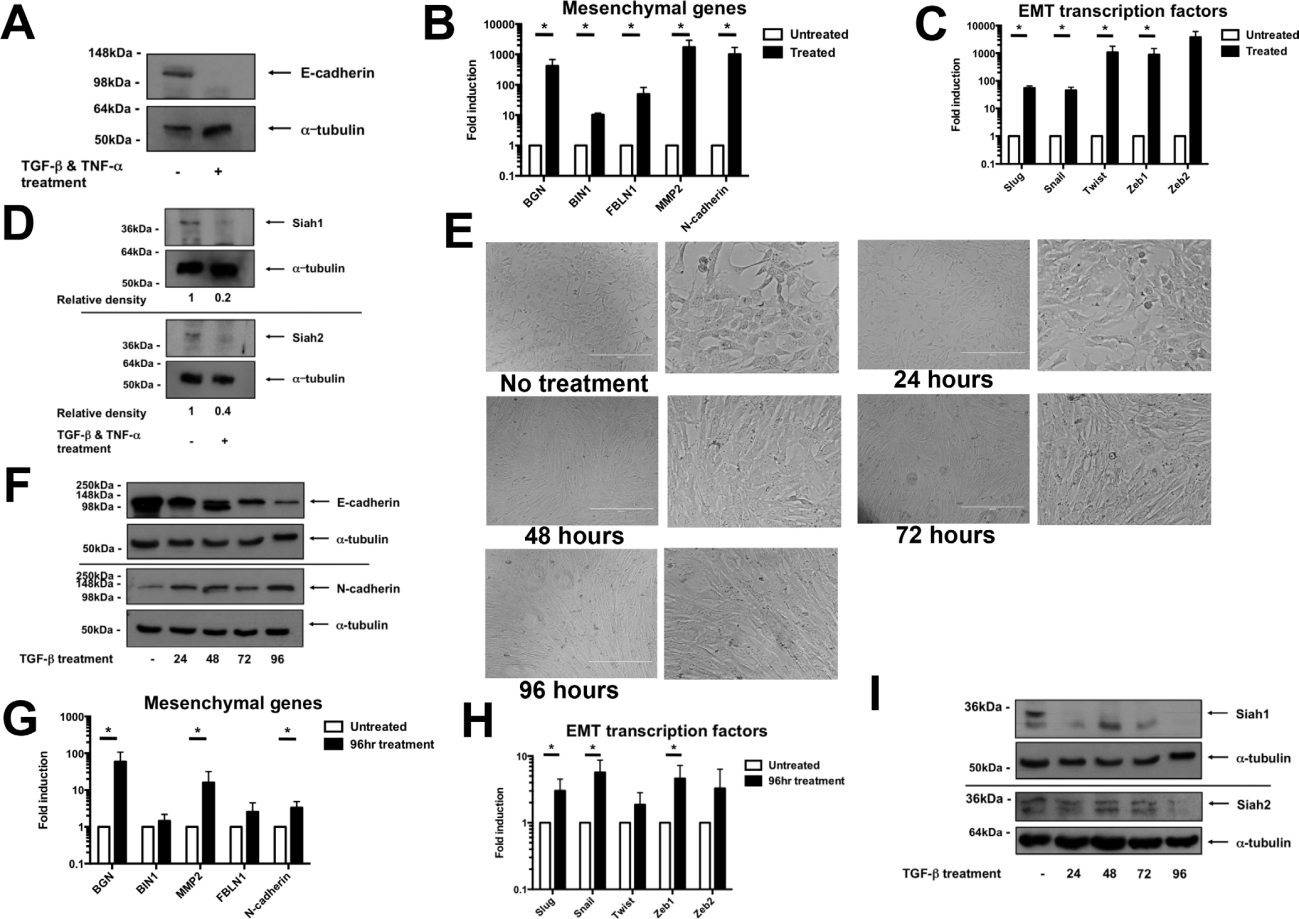
Siah protein levels decrease during EMT **(A)** Protein expression levels, as assessed by Western blot, of E-cadherin in MCF-7 untreated control cells and cells undergoing EMT, as induced by a combination of TGF-β and TNF-α treatment. Equal loading was confirmed by α-tubulin. **(B)** mRNA expression of mesenchymal genes in untreated control cells and treated MCF-7 cells. qPCR was performed in triplicate per biological repeat (*n* = 4). Data shown as mean ± SEM. **(C)** mRNA expression, as determined by qPCR performed in triplicate per biological repeat (*n* = 4), of EMT transcription factors in untreated and treated MCF-7 cells. Data shown as mean ± SEM. **(D)** Protein expression of Siah1 and Siah2 in untreated and treated MCF-7 cells, as evaluated by Western blot. Equal loading was confirmed by α-tubulin. Relative pixel density of Zeb1 was normalized to respective loading control. **(E)** Phase contrast photos of NMuMG cells treated with TGF-β over a 4-day period. Scale bar represents 400 μm. **(F)** Protein expression of E-cadherin and N-cadherin, as measured by Western blot, of NMuMG cells over the treatment period. Equal loading was confirmed by α-tubulin. **(G)** mRNA expression of mesenchymal genes of untreated control NMuMG cells and 96 hr treated cells. qPCR was performed in triplicate per biological repeat (*n* = 4). Data shown as mean ± SEM. **(H)** mRNA expression of EMT transcription levels as assessed by qPCR performed in triplicate per biological repeat (*n* = 4). Data shown as mean ± SEM. **(I)** Protein expression levels of Siah1 and Siah2, as assessed by Western blot, in NMuMG cells during TGF-β treatment regimen. Equal loading confirmed by α-tubulin.

To investigate Siah protein levels during EMT, a phenotypic switch in the human epithelial MCF-7 breast cancer cell line was induced by a treatment of TGF-β and TNF-α over a 5-day period. The EMT induction by this treatment regimen was confirmed by the complete loss of E-cadherin at the protein level in treated cells (Figure 1A). Additionally, mesenchymal gene expression (BGN, BIN1, FBLN1, MMP2 and N-cadherin) was significantly increased in treated cells (Figure 1B). Concurrently, EMT transcription factor gene expression (Slug, Snail, Twist, Zeb1, Zeb2) in treated cells was significantly increased (Figure 1C). Interestingly, the protein expression, but not gene expression, of both Siah family members was decreased following EMT induction (Figure 1D, Supplementary Figure 1A).

The reduction of Siah protein levels was also observed in the murine mammary epithelial cell line, NMuMG, when treated with TGF-β to induce EMT. NMuMG cells normally have an epithelial appearance, consisting of regular cellular dimensions and the formation of discrete epithelial islands. However, these cells adopted a mesenchymal phenotype, as characterized by elongated cell bodies and by growth as dispersed, single cells or in small groups, as early as 48 hours after the initiation of TGF-β treatment (Figure 1E). EMT induction was verified by the gradual decrease in E-cadherin and increase in N-cadherin protein expression, respectively, over the course of treatment (Figure 1F). This was also associated with increased expression of mesenchymal genes (Figure 1G, Supplementary Figure 1B). In addition, an increase in gene expression of EMT transcription factors was also observed in treated cells (Figure 1H, Supplementary Figure 1C). At the protein level, Siah1 and Siah2 expression was effectively reduced by 24 and 96 hours, respectively, following treatment initiation (Figure 1I). However, no such changes were observed at the gene expression level (Supplementary Figure 1D). This data shows that Siah protein levels are reduced in cells undergoing EMT.

### Siah knockdown leads to a mesenchymal gene expression profile

**Figure 2:**
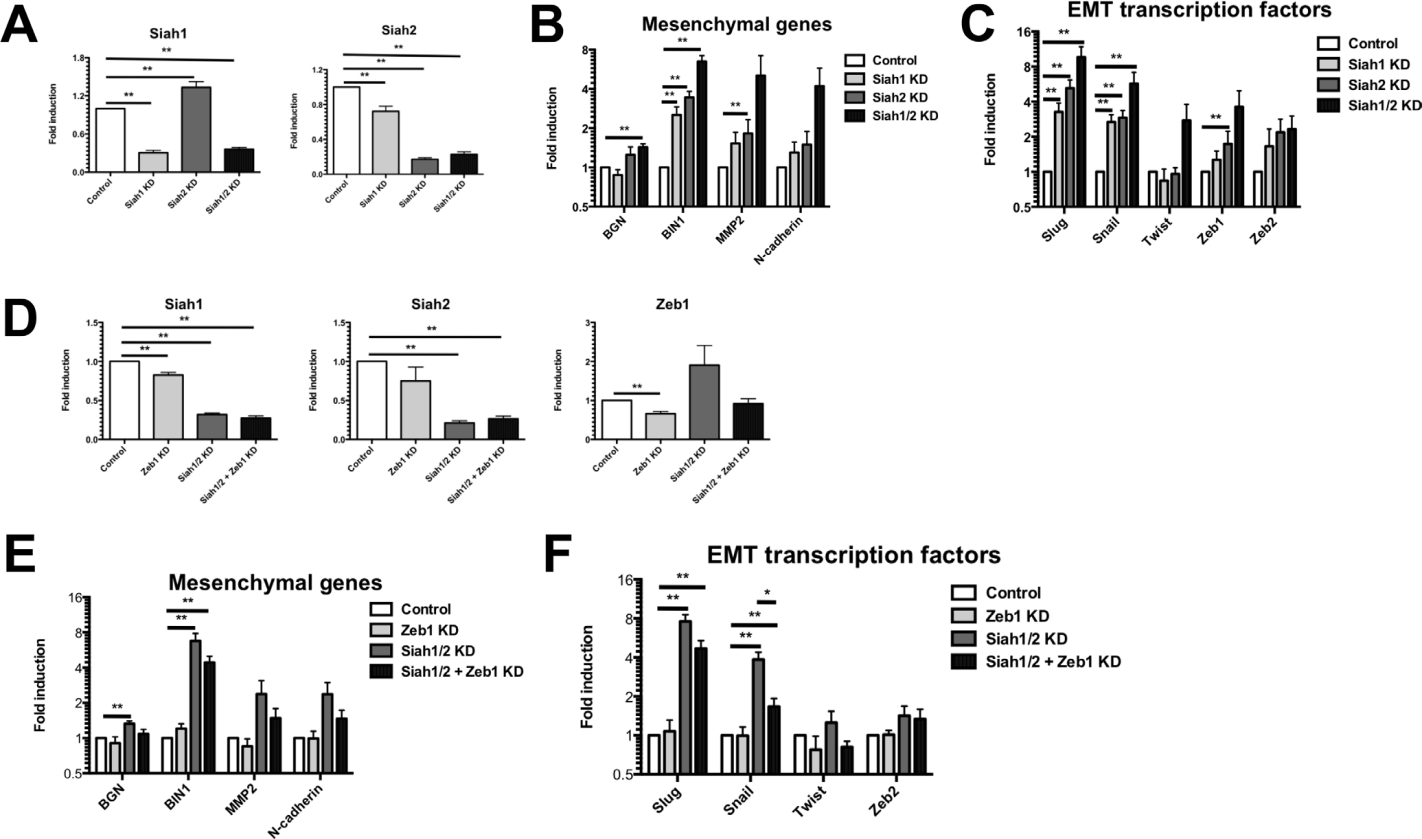
Siah knockdown induces an EMT gene profile **(A)** The efficiency of Siah knockdown with Dharmacon SMARTpool siRNAs as confirmed by qPCR. **(B)** mRNA expression of mesenchymal genes, following Siah1 and/or Siah2 knockdown, as assessed by qPCR. **(C)** The mRNA expression of EMT transcription factors, following Siah1 and/or Siah2 knockdown, as determined by qPCR. **(D)** Knockdown of Siah1, Siah2 and Zeb1 in MCF-7 cells knockdown with Dharmacon SMARTpool siRNAs. **(E)** mRNA expression of mesenchymal genes following knockdown of Zeb1 alone, Siah1 and Siah2 or Siah1, Siah2 and Zeb1. **(F)** The mRNA expression of EMT transcription factors following Zeb1, Siah1 and Siah2, and Siah1, Siah2 and Zeb1 knockdown. All knockdown qPCR reactions were performed in triplicate per biological repeat (*n* = 5). Gene expression levels are relative to control (set value of 1). Data shown as mean ± SEM.

To assess if a decrease of Siah has a functional role during EMT, Siah gene expression levels were reduced by siRNA-mediated knockdown in MCF-7 cells (Dharmacon SMARTpool; Figure 2A, Individual siRNAs; Supplementary Figure 2A). The knockdown of Siah1 and/or Siah2 was sufficient to up-regulate EMT target genes (Figure 2B, Supplementary Figure 2B) and transcription factors (Figure 2C, Supplementary Figure 2B), suggesting that the loss of Siah alone is able to initiate the induction of EMT. Interestingly, the knockdown of Siah1 led to an increase in Siah2 expression, indicative of a compensatory mechanism. While demonstrating lesser penetrance, similar trends in EMT target and transcription factor gene expression was observed when Siah gene expression levels were mediated by siRNA-knockdown (Dharmacon SMARTpool) in NMuMG cells (Supplementary Figure 2E, F). Next, to determine which EMT transcription factor mediated this specific induction of EMT, we focused on Zeb1 as it is the most proximal EMT transcription factor [6]. Thus, any alterations in upstream EMT transcription factors should manifest in changes of Zeb1 expression. To evaluate whether the loss of Siah modulates EMT through Zeb1, expression of Zeb1 was reduced by knockdown in combination with Siah1 and Siah2 knockdown (Figure 2D, Supplementary Figure 2C). The EMT gene signature caused by Siah1 and/or Siah2 knockdown (Figure 2E, Supplementary Figure 2D) was partially reverted by the reduction of Zeb1 in MCF-7 cells (Figure 2F, Supplementary Figure 2D). While in NMuMG cells, this pattern was only observed in N-cadherin gene expression, however, this is most likely caused by poor Zeb1 knockdown when combined with Siah1a, Siah1b and Siah2 siRNAs (Supplementary Figure 2G, H). Overall, this suggests that Siah controls EMT through a Zeb1-dependent process.

### Siah proteins target Zeb1 for proteasomal degradation

**Figure 3:**
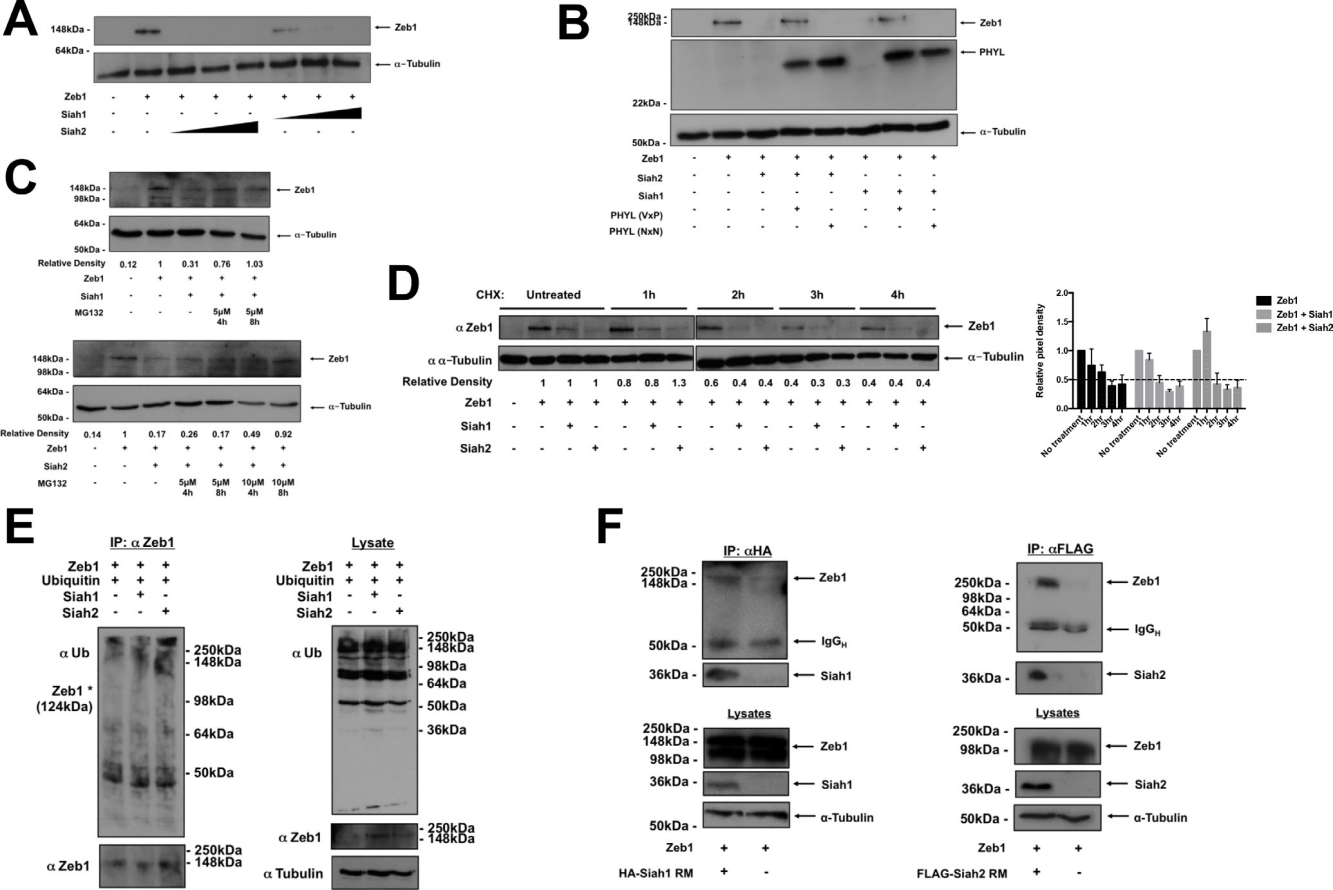
Siah causes reduction of Zeb1 protein levels **(A)** Western blot of Zeb1 protein abundance in U2OS cells expressing Zeb1 alongside increasing concentrations of Siah1 or Siah2 (50 ng, 250 ng, 500 ng) as indicated. Equal loading was confirmed by α-tubulin. **(B)** Siah1, Siah2, Zeb1, PHYL and PHYL NxN were expressed in U2OS cells, as indicated. Following transfection, Western blot was performed to determine protein expression of Zeb1 and PHYL (via HA-tag expression). Equal loading was confirmed by α-tubulin. **(C)** Siah1, Siah2 and Zeb1 were expressed in cells and then treated with MG132, at concentrations and for time periods as indicated. Zeb1 protein expression was then assessed by Western blot. Loading was determined by α-tubulin. **(D)** Siah1, Siah2 and Zeb1 were expressed in U2OS cells and then treated with 100 μg/ml cycloheximide (CHX) for time periods as indicated. Zeb1 protein expression was then assessed by Western blot. Loading was determined by α-tubulin. Relative pixel intensities of Zeb1 are represented by the mean values of 3 independent experiments. The adjacent graph shows mean relative pixel densities ± SEM of 3 independent experiments. **(E)** Siah1, Siah2, Zeb1 and ubiquitin was expressed in U2OS cells as indicated. Cells were then incubated with MG132 and to measure protein ubiquitylation, immunoprecipitation of Zeb1 was performed. Ubiquitin and Zeb1 expression was analyzed by Western blot. Loading was determined by α-tubulin. **(F)** Co-immunopreciptation of Zeb1 with Siah1 RING mutant (left) and Siah2 RING mutant (right). 10% of cell lysates were used for analysis of input.

Siah proteins are ubiquitin ligases that regulate signal transduction by targeting substrates for proteasomal degradation [16]. To examine whether Siah proteins could alter Zeb1 protein levels, Zeb1 was overexpressed alongside increasing concentrations of Siah1 or Siah2 in the human osteosarcoma cell line, U2OS. Expression of either Siah1 or Siah2 was associated with decreased Zeb1 protein abundance (Figure 3A, Supplementary Figure 3A). We have previously identified a peptide inhibitor of Siah called Phyllopod (PHYL), which blocks Siah-mediated degradation by preventing substrate interaction with Siah [24, 33]. PHYL, but not the mutant form of PHYL (PHYL NxN) that is unable to bind to Siah, inhibited Siah-mediated degradation of Zeb1 (Figure 3B, Supplementary Figure 3B). In addition, the proteasome inhibitor MG132 was capable of preventing the Siah-mediated reduction of Zeb1, demonstrating the importance of the proteasome in this process (Figure 3C). The half-life of Zeb1 is three hours, as assessed by cycloheximide (CHX) treatment (Figure 3D). In the presence of Siah1 or Siah2, the half-life of Zeb1 was reduced to two hours (Figure 3D). However, when co-transfected with the E3-ligase deficient RING mutant (RM) form of Siah1, the half-life of Zeb1 returns to 3 hours (Supplementary Figure 3C). The RM form of Siah2, however, prolongs the half-life of Zeb1 to beyond 4 hours. These mutant forms of Siah are able to bind to substrates, but due to mutations in their RING regions, are unable to transfer ubiquitin to substrates. To examine the induction of Zeb1 ubiquitylation by Siah proteins, Siah1-RM or Siah2-RM were co-expressed with ubiquitin and Zeb1 in U2OS cells, in the absence or presence of MG132 (Figure 3E). These results demonstrate that in the presence of Siah1 or Siah2, there is increased Zeb1 ubiquitylation. We next sought to determine whether Siah1 and Siah2 interact with Zeb1 by co-immunoprecipitation with the RING mutant forms of Siah1 and Siah2. Using this approach, Zeb1 was found to co-precipitate with Siah1 and Siah2, showing that Siah proteins and Zeb1 are binding partners (Figure 3F). Together, these results show that both Siah1 and Siah2 interact with and target Zeb1 for proteasomal degradation.

### Siah2 knockout causes a mesenchymal phenotype in mammary carcinoma cell lines

**Figure 4:**
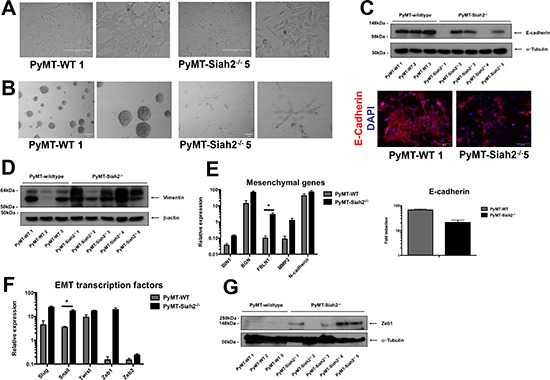
PyMT-Siah2^−/−^ cell lines undergo spontaneous EMT **(A)** Phase contrast images of PyMT-WT and PyMT-Siah2^−/−^ cells in 2D culture. Two representative images shown. Scale bar represents 400 μm. **(B)** Morphology of PyMT-WT and PyMT-Siah2^−/−^ cells in 3D Matrigel-embedded culture (two representative images shown, scale bar represents 100 μm). **(C)** Protein expression of E-cadherin cadherin in PyMT-WT and PyMT-Siah2^−/−^ cell lines, as determined by Western blot (left panel), and intracellular localization as shown by immunofluroescence (right panel; two representative images shown, scale bar represents 100 μm). **(D)** Vimentin protein expression of PyMT-WT and PyMT-Siah2^−/−^ cells as determined by Western blot. Equal loading was confirmed by β-actin. **(E)** mRNA expression of mesenchymal genes and E-cadherin in PyMT-WT and PyMT-Siah2^−/−^ cells as determined by qPCR. All qPCR reactions were performed in triplicate per technical repeat (three technical repeats). Data shown as mean ± SEM. **(F)** The mRNA expression of EMT transcription factors in PyMT-WT and PyMT-Siah2^−/−^ cells as determined by qPCR. All qPCR reactions were performed in triplicate per technical repeat (three technical repeats). Data shown as mean ± SEM. **(G)** Zeb1 protein levels of PyMT-WT and PyMT-Siah2^−/−^ cells were assessed by Western blot. Equal loading was confirmed by α-tubulin.

To ascertain the functional consequences of genetic loss of Siah in breast cancer, we utilized the Polyoma Middle T (PyMT)-driven mouse model of breast cancer in a Siah2^−/−^ background, as previously described [34]. Three independent wildtype (PyMT-WT) and five independent Siah2^−/−^ (PyMT-Siah2^−/−^) breast cancer cell lines were isolated from tumors that originated from different mice [34]. Cells were sorted for EpCAM positivity preceding isolation to exclude fibroblast contamination. Assessing the morphology of PyMT-Siah2^−/−^ cell lines in culture, we observed a mesenchymal phenotype (Figure 4A, Supplementary Figure 4A). In contrast, PyMT-WT cell lines possessed a typical epithelial morphology (Figure 4A, Supplementary Figure 4A). Similarly, in three-dimensional cell culture, PyMT-WT cells formed spheres without apparent invasion into surrounding matrix (Figure 4B, Supplementary Figure 4B). Two of the five PyMT-Siah2^−/−^ cell lines, however, exhibited an invasive phenotype, as evidenced by the branching acini, in three-dimensional matrices (Figure 4B, Supplementary Figure 4B). These data show that PyMT-Siah2^−/−^ cell lines have a more mesenchymal phenotype.

Importantly, there was a decrease in E-cadherin gene expression in the PyMT- Siah2^−/−^ cell lines (Figure 4C). At the protein level, all three PyMT-WT cell lines were positive for E-cadherin protein expression, while PyMT-Siah2^−/−^ cell lines either had a decreased, or lacked, expression of E-cadherin (Figure 4C, Supplementary Figure 4C). Interestingly, all cell lines were positive, albeit to different degrees, for N-cadherin protein expression (Supplementary Figure 4D). Also, there was overall a stronger expression of Vimentin, an EMT marker, in PyMT-Siah2^−/−^ cell lines compared to their wildtype counterparts (Figure 4D). In order to gauge whether the morphological differences observed in PyMT-Siah2^−/−^ cell lines were associated with a change in mesenchymal gene expression, the mRNA abundance of BIN1, BGN, FBLN1 and MMP2 was measured. Firstly, Siah expression was evaluated in these cell lines. While there were varying levels of Siah1a and Siah1b gene expression in all cell lines, the PyMT-Siah2^−/−^ cells lines, as expected, lacked Siah2 gene expression (Supplementary Figure 4E). Accordingly, PyMT-Siah2^−/−^ cell lines showed increased expression of these mesenchymal genes compared to PyMT-WT cell lines (Figure 4E, Supplementary Figure 4F). Overall, there was a decrease in E-cadherin gene expression (Figure 4E, Supplementary Figure 4F). Similarly, the expression of EMT transcription factors was increased in PyMT-Siah2^−/−^ cell lines (Figure 4F, Supplementary Figure 4G).

Having shown that Zeb1 is a substrate of Siah ubiquitin ligase function (Figure 3), we therefore expected increased protein expression of Zeb1 in the PyMT-Siah2^−/−^ cell lines. Zeb1 protein was detected in four of the five PyMT-Siah2^−/−^ cell lines, whereas all three PyMT-WT cell lines were negative for Zeb1 (Figure 4G). Together, these data demonstrate that the genetic loss of Siah2 causes sustained EMT *in vitro*, resulting in a mesenchymal phenotype that is associated with an increase of Zeb1 protein abundance.

## DISCUSSION

**Figure 5:**
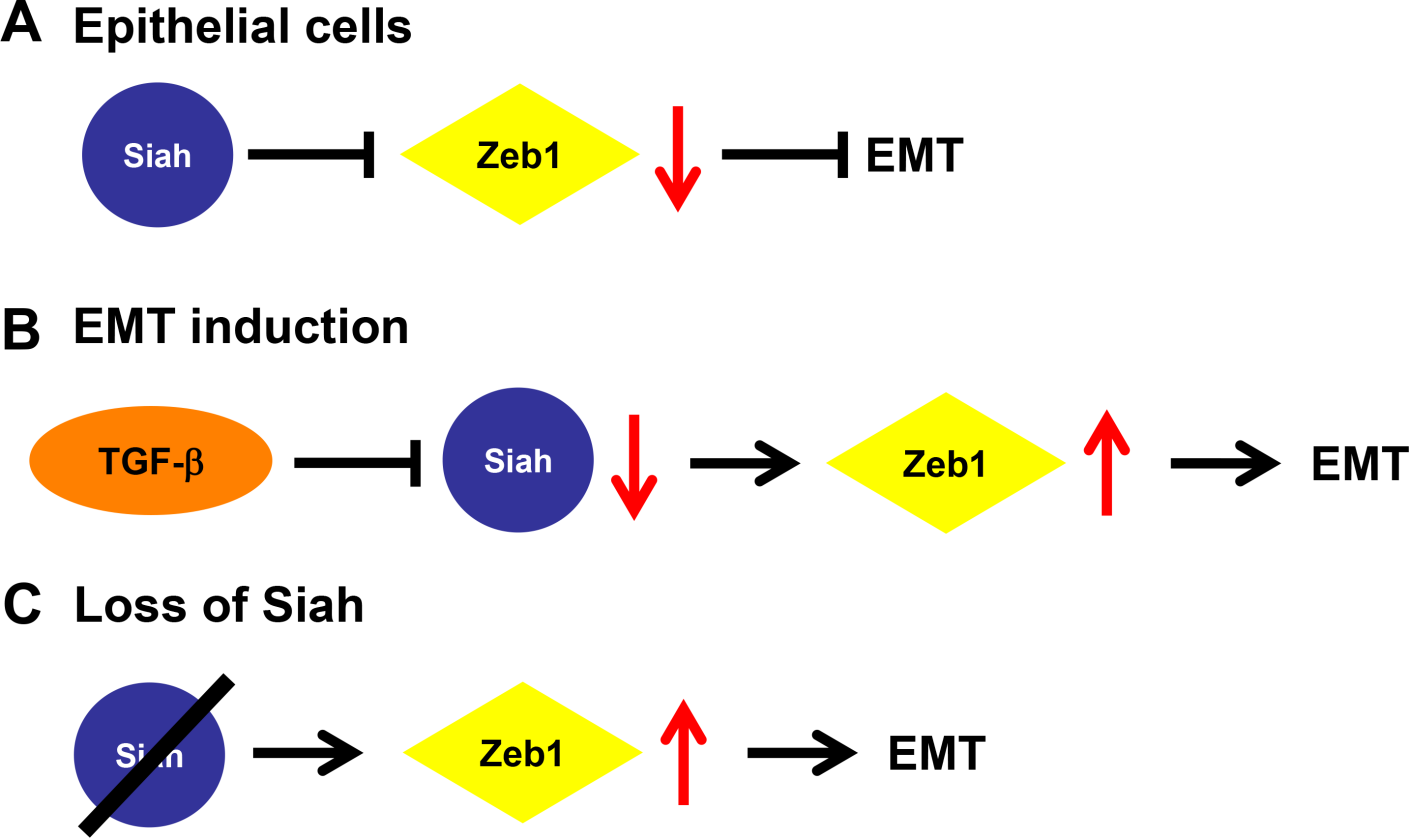
Proposed mechanism of Siah-mediated regulation of EMT **(A)** In epithelial cells, Siah expression maintains Zeb1 protein at low levels, thereby preventing EMT induction. **(B)** With active TGF-β signaling, Siah expression is down-regulated, leading to an increase of Zeb1 protein abundance and the induction of the EMT program **(C)** With the loss of Siah, Zeb1 levels are unchecked and are then able to induce EMT.

Currently, the post-translational regulatory mechanisms of Zeb1 are not well understood. Here we show that Siah proteins, a family of E3 ubiquitin ligases, are novel regulators of Zeb1 and therefore, EMT. Taken together, these data indicate that in epithelial cells, the presence of Siah maintains Zeb1 protein at low levels, thus preventing the induction of EMT (Figure 5A). However, with active TGF-β signaling, Siah is down-regulated, which then allows Zeb1 protein levels to increase, thereby inducing EMT (Figure 5B). Similarly, with the loss of Siah, Zeb1 protein levels accumulate and induce EMT (Figure 5C).

These data demonstrate that while the PyMT-Siah2^−/−^ cell lines were generally more mesenchymal than the PyMT-WT cell lines, there was strong heterogeneity amongst the different lines. In fact, the cell line we had previously used, PyMT-Siah2^−/−^ 2, was the most epithelial of the PyMT-Siah2^−/−^ cell lines and readily grew tumours in mice [34]. Furthermore, as Siah1 can also regulate Zeb1 and is itself regulated during EMT, varying levels of compensatory up-regulation of Siah1a and/or Siah1b at the protein level may be able to partially rescue the PyMT-Siah2^−/−^ cell lines from a mesenchymal phenotype. However, it is possible that other factors may be regulating Siah during the EMT program. Additionally, as part of the purification process, cells only expressing EpCAM, an epithelial cells marker, were selected following cell isolation. This suggests that loss of Siah is able to naturally induce an epithelial-to-mesenchymal drift in a population that was initially purely epithelial. It is clear that the PyMT-WT cell lines are epithelial, and while there is variability amongst the PyMT-Siah2^−/−^ cell lines, there is clearly a strong trend towards a mesenchymal phenotype.

TGF-β is known to be the primary upstream inducer of EMT, yet how multiple signaling pathways interact with one another to activate EMT remains unclear. Previous work has identified two possible methods of hypoxia-mediated Siah activation: phosphorylation by p38 MAPK and gene transcription induction through Akt signaling [19]. Interestingly, both of these well-known pathways have been shown to be targets of TGF-β [35, 36]. It is possible that Siah expression is modulated by TGF-β via both or either of these pathways. In addition to this, Siah is known to interact with the Ras pathway and β-catenin, both of which are also downstream targets of TGF-β signaling and associated with EMT [37–39]. Further work should focus on delineating the precise pathways through which TGF-β is acting upon Siah.

It is becoming increasingly clear that Siah may have a dual role, as both a tumor suppressor and an oncogene in cancer [17, 40]. Our work suggests that Siah may be acting as a tumor suppressor in the context of EMT and metastasis via suppression of Zeb1. By targeting Zeb1 for degradation, Siah may be able to prevent the induction or interrupt the persistence of EMT. By doing so, Siah may prevent tumor cells from disseminating from the primary tumor, thus inhibiting metastasis. EMT is also known to be a vital process during embryonic development. Interestingly, Siah1a/Siah2 double knockout mice are embryonic lethal and Siah2 has been shown to be expressed at particular sites during development [41, 42]. This demonstrates a level of functional redundancy between Siah2 and Siah1a *in vivo*, but is a possible indicator of the importance of Siah in controlling EMT, particularly during early development. The loss of both Siah1a and Siah2 may interrupt the activation of EMT during the developmental process, causing the death of these mice at the embryonic stage.

Considering the importance of Zeb1 in EMT initiation, the findings presented here have implications on our current understanding of the TGF-β signaling network, the regulation of Zeb1 and the molecular steps behind the aberrant induction of the EMT program in cancer. This work highlights the complexity and dynamism of EMT regulation and the need for a greater comprehension of how EMT is induced and its importance in the cancer setting. Understanding the mechanisms of tumor cell escape, survival and their subsequent colonization and outgrowth in secondary organs will aid in the development of novel treatments for metastatic cancer.

## MATERIALS & METHODS

### Primary cell isolation, cell culture and various assays

PyMT-derived tumor cell lines were generated as previously described [34]. Cell culture with MCF-7, U2OS, PyMT-WT and PyMT-Siah2^−/−^ cells lines was performed as previously published [24, 34]. NMuMG cells were maintained in Dulbecco's modified Eagle's medium, containing 10% (v/v) fetal calf serum, 1% (w/v) penicillin/streptomycin and 0.2% (v/v) insulin. EMT was induced in MCF-7 cells by a 5-day daily treatment of TGF-β (R&D Systems) and TNF-α (R&D Systems) at 2 ng/ml and 10 ng/ml, respectively. NMuMG cells were treated with 5 ng/ml of TGF-β (R&D Systems) daily, over several different time-points (1–4 days). Cells were then harvested for gene and protein expression analysis.

For transient siRNA-mediated knockdown, MCF-7 cells were seeded at a density of 1.3 × 10^5^ cells/well, while NMuMG cells were seeded at a density of 7000 cells/well, in 12-well plates the day prior to trans-fection. DharmaFECT1 (ThermoScientific) and siRNAs (Dharmacon SMARTpool and individual siRNAs from the SMARTpool; ThermoScientific) were pre-incubated for 15 minutes at room temperature in serum-free media. Then, cells were incubated in a final transfection solution, comprising of 0.4% (v/v) DharmaFECT 1 and 50 nM siRNA in antibiotic-free media. After 24 hours, media was replaced and following a further 48 hours, cells were harvested for gene expression analysis. For transient overexpression assays, cells were transfected with expression plasmids using Lipofectamine 2000 (Invitrogen) according to the manufacturer's instructions and as previously described [24]. Expression plasmids for Siah1, Siah2, Phyllopod VxP, Phyllopod NxN and Zeb1 have been described [24, 43]. To inhibit proteasomal activity, transfected cells were treated with MG132 (Merck Millipore) as indicated. To inhibit protein synthesis, transfected cells were treated with cycloheximide (Sigma-Aldrich) as indicated.

To visualize the growth of cells on plastic (2-dimensional growth), phase contrast images of cells were taken with the Evos microscope (Advanced Microscopy Group). To observe cell growth in 3-dimensional suspension, cells were seeded in a mix of media and 2% Matrigel (BD Laboratories), in duplicate, on a 40 μl Matrigel bed. These cells were seeded at 3000 cells per chamber in a 8-well chamber slide (Thermo Fischer Scientific). Images of cells in culture were taken four days later with an inverted microscope (Leica).

### Co-immunoprecipitation

U2OS cells were transfected as above. Following recovery, cells were lysed in NP40 buffer containing 10 ug/ml Aprotinin and 10 ug/ml Pepstatin. Co-immunoprecipitation was performed as previously published [18]. Protein ubiquitylation was measured by culturing transfected cells with 5 μM MG132 for 10 hours and then lysing in NP40 buffer as above.

### Western blotting

Cells were lysed and analyzed by Western blotting as described previously [24]. Primary antibodies used are as follows: Flag, α-tubulin, β-actin (Sigma-Aldrich); E-cadherin, N-cadherin (BD Laboratories); Siah1 (as previously described [44]); Siah2 (Novus Biologicals); Siah2, Zeb1 (Santa Cruz Biotechnology); Vimentin, Zeb1 (Cell Signaling Technology); HA (a kind gift from Rick Pearson, Peter MacCallum Cancer Centre). To quantify Western blots, densities of bands were measured in Image J [45]. These values were then normalized to the densities of their corresponding loading control. In cycloheximide treatment experiments, values of samples were then normalized to the positive control.

### mRNA and qRT-PCR

mRNA was extracted using the QiaShredder kit (Qiagen) and the RNeasy Plus Mini Kit (Qiagen) according to the manufacturer's instructions. qRT-PCR was performed with converted cDNA as previously published [34]. Results were normalized to housekeeping genes CETN2 (human), and EF-1 or HMBS (mouse). Sequences (5′ to 3′) of primers (GeneWorks) used are as follows:

**Table:** 

Mouse EF-1	Sense: CATCAACATCGTCGTAATCGGA Antisense: CTTGTCGA TTCCGCCACA T
Mouse HMBS	Sense: GTGTTGCACGATCCTGAAACT Antisense: GTTGCCCATCCTTTATCACTGTA
Mouse E-cadherin	Sense: CAGGTCTCCTCA TGGCTTTGC Antisense: CTTCCGAAAAGAAGGCTGTCC
Mouse N-cadherin	Sense: AGCGCAGTCTTACCGAAGG Antisense: TCGCTGCTTTCATACTGAACTTT
Mouse Siah1a	Sense: AAGTGTCCACCA TCCCAGAG Antisense: ATGTAAGTTTGGGGCGACAG
Mouse Siah1b	Sense: GCTACAGCATTATCCACTGGC Antisense: AGGACACTCAAAAAGACTCGC
Mouse Siah2	Sense: CCAATGCCGCCAGAAGTTAAG Antisense: CAGGGAAACAGAACTGCCGA
Mouse BIN1	Sense: AGGATCTTCGGACCTATCTGGC Antisense: GGCTTCGTGCATCGCTTTAAC
Mouse TBX3	Sense: TGGAACCCGAAGAAGACGTAG Antisense: TACCCCGCTTGTGAAACTGG
Mouse FBLN1	Sense: ATCAGATGGCTAACCAGCACA Antisense: A TCCTGCACTCCTTGGA TTCT
Mouse MMP2	Sense: TTCTGGTCAAGGTCACCTGTC Antisense: CAAGTTCCCCGGCGA TGTC
Mouse Zeb1	Sense: GCTGGCAAGACAACGTGAAAG Antisense: GCCTCAGGATAAATGACGGC
Mouse Zeb2	Sense: AGGCTCGGAGACAGA TGAAGA Antisense: GCGGACAGACAGACACTTACC
Mouse Twist	Sense: GGACAAGCTGAGCAAGA TTCA Antisense: CGGAGAAGGCGTAGCTGAG
Mouse Slug	Sense: TGGTCAAGAAACA TTTCAACGCC Antisense: GGTGAGGATCTCTGGTTTTGGTA
Mouse Snail	Sense: CACACGCTGCCTTGTGTCT Antisense: GGTCAGCAAAAGCACGGTT
Human CETN2	Sense: CGGACTCCTTTGGCTATGGCCTC Antisense: TGGTGCCAGTTCCATCCGCA
Human BGN	Sense: CAGTGGCTTTGAACCTGGAG Antisense: CAGCTTGGAGTAGCGAAGCA
Human BIN1	Sense: CGATCTTGTTTGCCTCATCCC Antisense: TGAGCAGTGCGTCCAGAATTT
Human FBLN1	Sense: AGAGCTGCGAGTACAGCCT Antisense: AAGACCTGTCCACACTGGTAG
Human MMP2	Sense: TACAGGATCATTGGCTACACACC Antisense: GGTCACATCGCTCCAGACT
Human N-cadherin	Sense: ATCGCATTATGCAAGACTGGATT Antisense: ATGCACATCCTTCGATAAGACTG
Human Slug	Sense: AAGCATTTCAACGCCTCCAAA Antisense: GGATCTCTGGTTGTGGTATGACA
Human Snail	Sense: TCGGAAGCCTAACTACAGCGA Antisense: AGATGAGCATTGGCAGCGAG
Human Twist	Sense: GTCCGCAGTCTTACGAGGAG Antisense: GCTTGAGGGTCTGAATCTTGCT
Human Zeb1	Sense: GATGATGAATGCGAGTCAGATGC Antisense: ACAGCAGTGTCTTGTTGTTGT
Human Zeb2	Sense: CAAGAGGCGCAAACAAGCC Antisense: GGTTGGCAATACCGTCATCC
Human Siah1	Sense: TAAATGGTCATAGGCGACGA Antisense: GCAATGCTGGTGTCAAAGAC
Human Siah2	Sense: AATCACCCGGAGTGCTTATC Antisense: GGACCCTTTCCCACAATTTA

### Immunoflourescence

PyMT-WT and PyMT-Siah2^−/−^ cells were seeded on glass coverslips in a 12-well plate at subconfluent levels and incubated for 24 hours to adhere. Plates were then fixed with methanol and stained with E-cadherin or N-cadherin antibodies (BD Laboratories) according to manufacturer's instructions. Slides were observed with the Olympus BX-51 or BX-61 microscope (Olympus).

### Statistical analysis

For gene expression comparison in MCF-7 and NMuMG untreated control and treated cells, results were normalized to untreated control cells. The Wilcoxon rank test was utilized in R to determine statistical significance. Data is displayed on a log10 scale. To determine significance in non-normalized results, the unpaired, two-tailed Student's t-test was employed. For the analysis of siRNA-mediated knockdowns, the one sample t-test, with the theoretical mean set as 1, was employed when comparing knockdown groups to control. Data is displayed on a linear (Siah1 KD, Siah2 KD, Zeb1 KD) or a log2 scale. When comparing gene expression levels between PyMT-WT and PyMT-Siah2^−/−^ cell lines, the Mann-Whitney test was used. Data is displayed on a log10 scale. Statistical significance was denoted as follows: *p < 0.05, **p < 0.01, ***p < 0.001. Data shown is mean ± SEM unless indicated otherwise.

## SUPPLEMENTARY FIGURES


