# Supplementary material for: The ubiquitin ligase Siah is a novel regulator of Zeb1 in breast cancer
Source: Oncotarget. 2014 Dec 18;6(2):862–73. doi: 10.18632/oncotarget.2696 (PMC4359261; doi:10.18632/oncotarget.2696)
Supplement: Supplementary file 1 [file oncotarget-06-862-s001.pdf]

SUPPLEMENTARY FIGURES

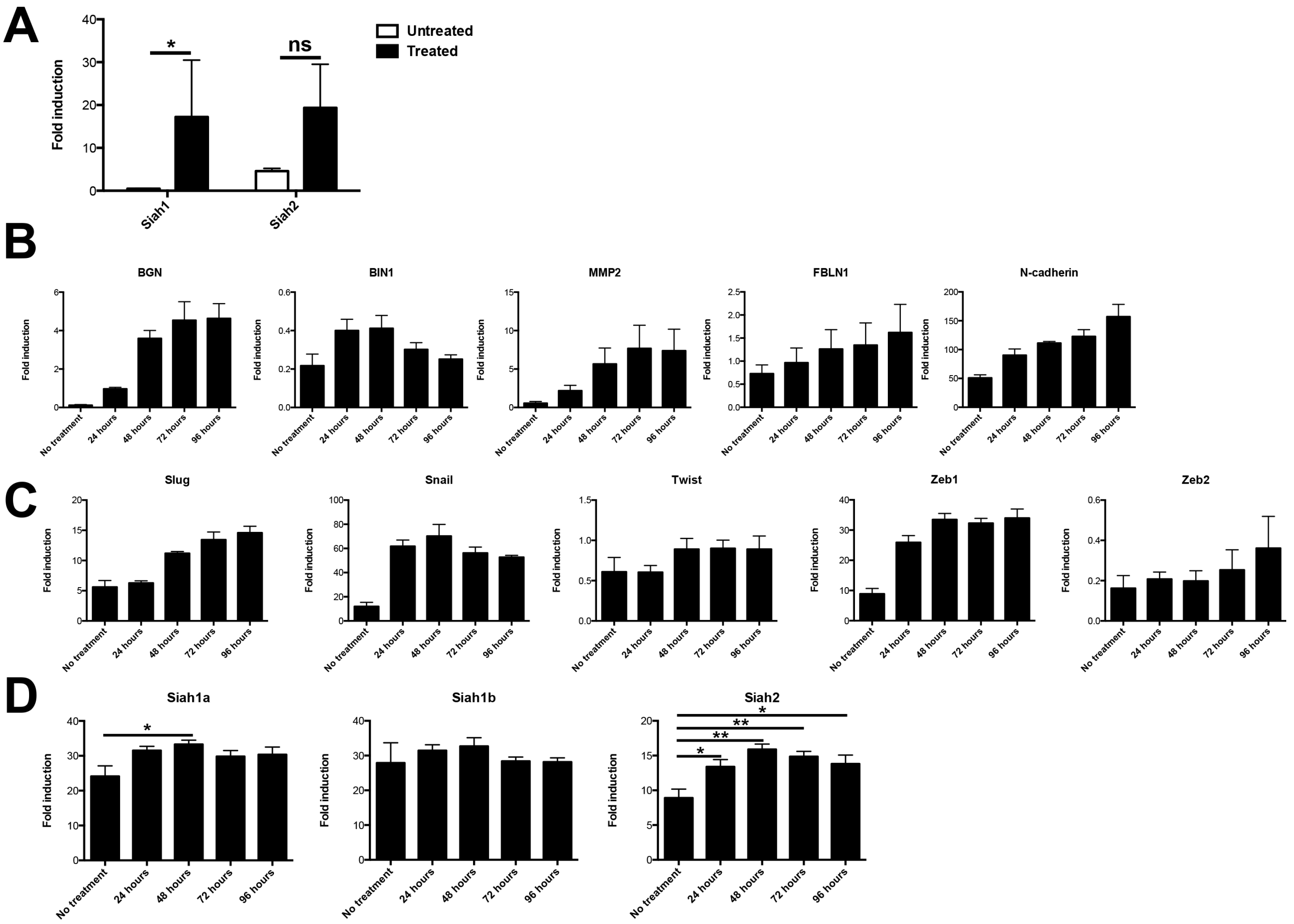

Supplementary Figure S1: Gene expression profiles of NMuMG cells during TGF-β treatment. **(A)** The mRNA expression levels of Siah in MCF-7 untreated and treated cells, as measured by qPCR. **(B)** The mRNA expression level of mesenchymal genes in NMuMG cells during TGF-β treatment, as assessed by qPCR. **(C)** mRNA expression levels of EMT transcription factors during TGF-β treatment, as measured by qPCR. **(D)** The mRNA expression of Siah1a, Siah1b and Siah2 during the 96-hour TGF-β treatment period as determined by qPCR. All qPCRs were performed in triplicate per biological repeat ($n = 4$). Data shown as mean ± SEM.

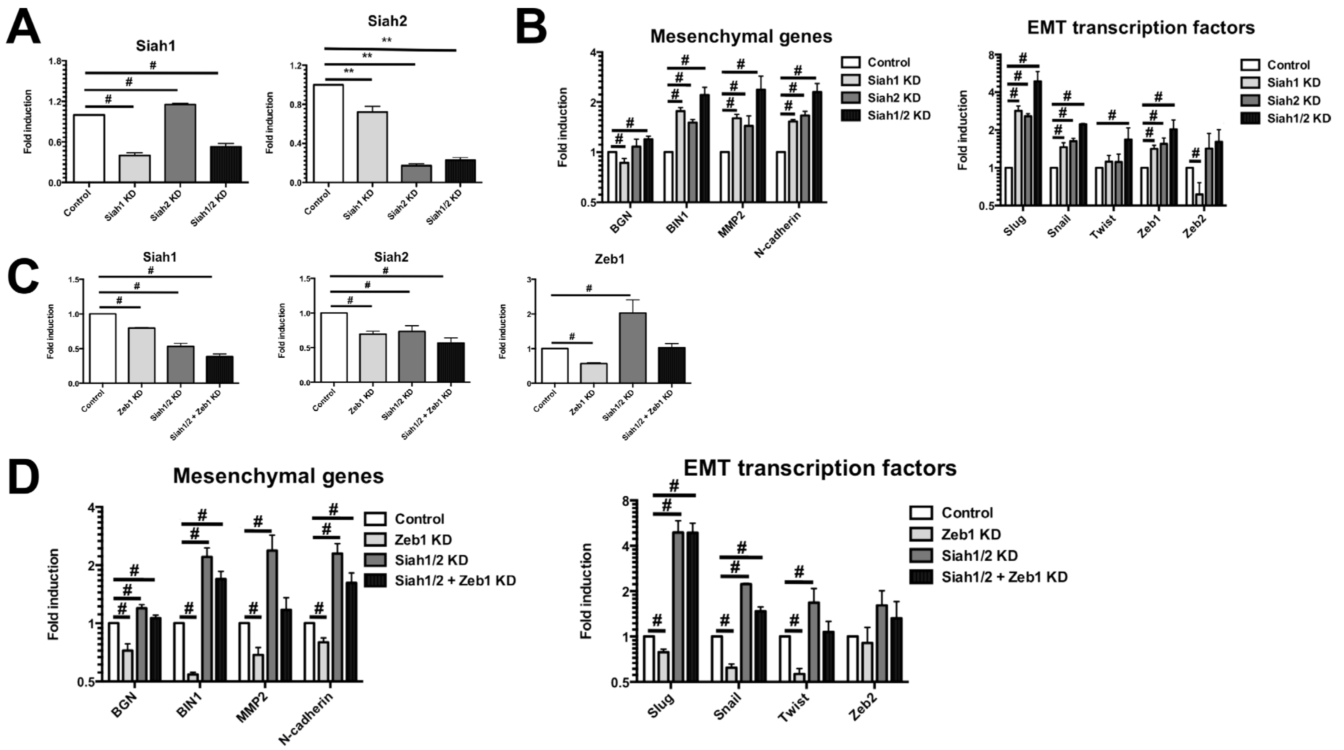

Supplementary Figure S2: Siah knockdown induces an EMT gene profile. (A) The knockdown efficiency of Siah1 and Siah2 with individual Dharmacon siRNAs in MCF-7 cells, as measured by qPCR. **(B)** mRNA expression, as assessed by qPCR, of EMT target genes and EMT transcription factors, following Siah1 and/or Siah2 knockdown. **(C)** Knockdown efficiency of Siah1, Siah2 and Zeb1 in MCF-7 cells with individual Dharmacon siRNAs in MCF-7 cells, as determined by qPCR. **(D)** mRNA expression of EMT target genes and transcription factors following Zeb1, Siah1 and Siah2, and Siah1, Siah2 and Zeb1 knockdown with individual Dharmacon siRNAs. All knockdown qPCR reactions were performed in triplicate per biological repeat ($n = 3$). Gene expression levels are relative to control (set value of 1). # represents $p = 0.06$. Data shown as mean ± SEM.

(*Continued*)

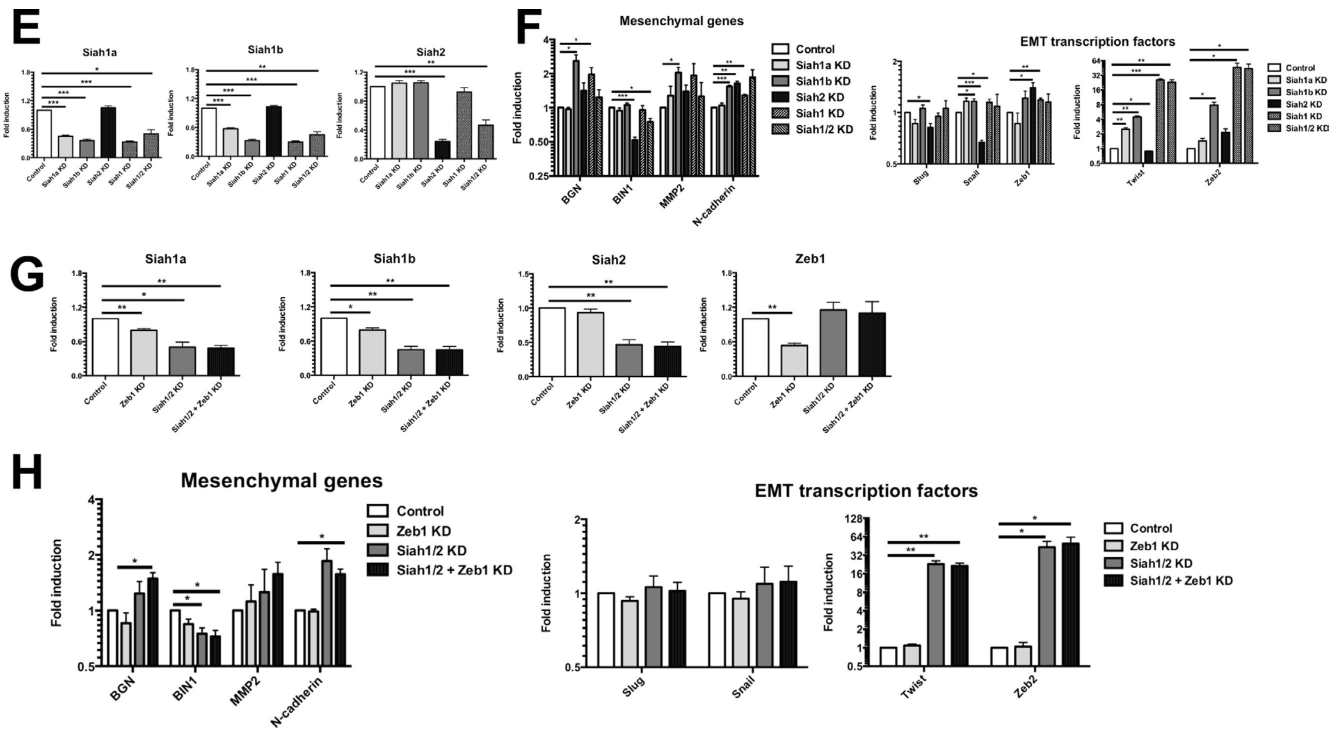

Supplementary Figure S2 (*Continued*): (E) The knockdown efficiency of Siah1a, Siah1b and Siah2 with Dharmacon SMARTpool siRNAs in NMuMG cells, as confirmed by qPCR. **(F)** The mRNA expression of EMT target genes and EMT transcription factors, as measured by qPCR, following Siah1a, Siah1b and/or Siah2 knockdown. **(G)** Knockdown efficiency of Siah1, Siah2 and Zeb1 in NMuMG cells with Dharmacon SMARTpool siRNAs in NMuMG cells, as assessed by qPCR. **(H)** The mRNA expression of EMT target genes and transcription factors following Zeb1, Siah1 and Siah2, and Siah1, Siah2 and Zeb1 knockdown with Dharmacon SMARTpool siRNAs in NMuMG cells. All knockdown qPCR reactions were performed in triplicate per biological repeat (n = 4). Gene expression levels are relative to control (set value of 1). Data shown as mean ± SEM.

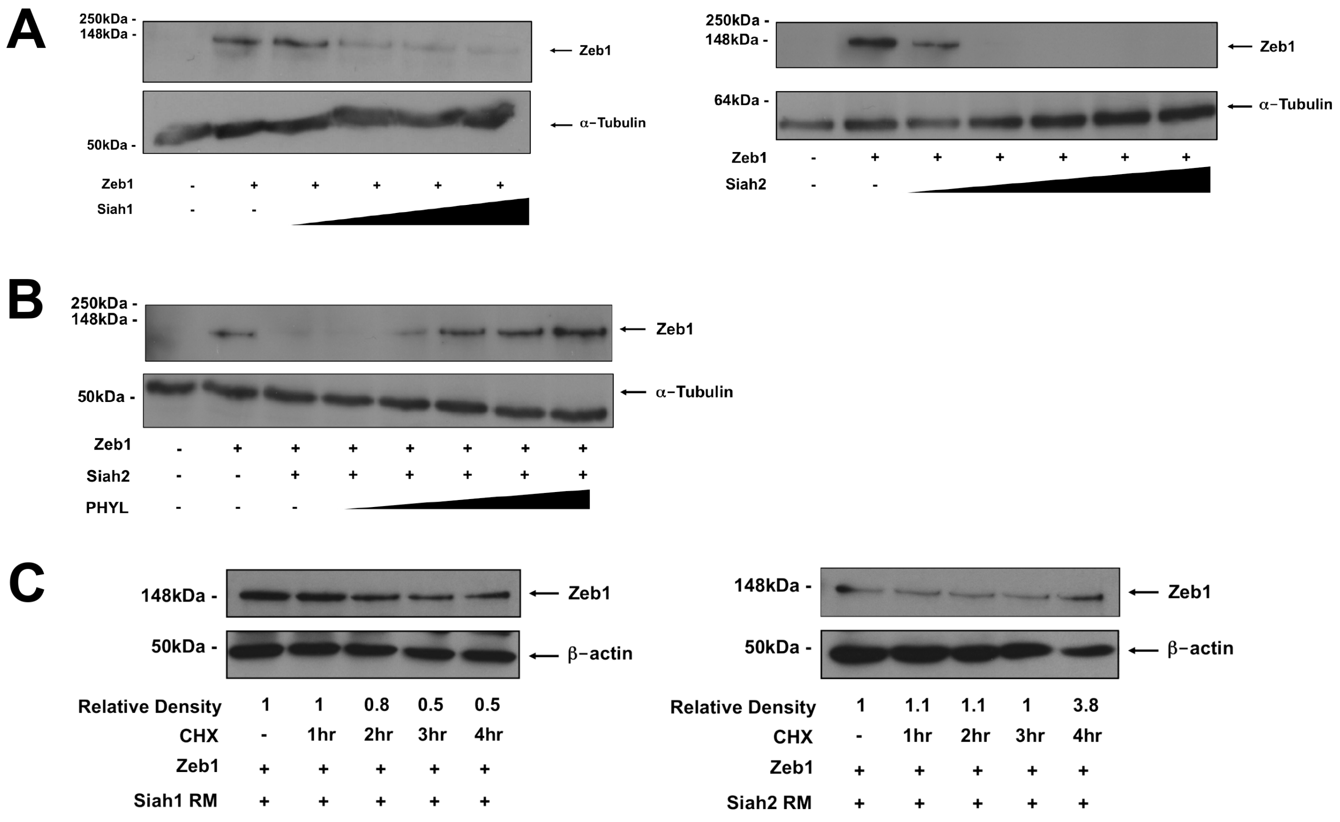

Supplementary Figure S3: Siah-mediated degradation of Zeb1 can be blocked by PHYL. **(A)** Co-expression of Zeb1 accompanied by increasing concentrations of Siah1 (5 ng, 15 ng, 20 ng, 25ng) and Siah2 (1 ng, 5 ng, 10 ng, 15 ng, 20 ng) to determine the minimum concentration needed to effectively reduce Zeb1 protein abundance, as assessed by Western blot. Equal loading was confirmed by α-tubulin. **(B)** Co-expression of Zeb1, Siah2 and increasing concentrations of PHYL (1 ng, 5 ng, 10 ng, 25 ng, 50 ng) to determine minimum concentrated needed to effectively prevent Siah2-meditated Zeb1 reduction, as determined by Western blot. Equal loading was confirmed by α-tubulin. **(C)** Siah1-RM, Siah2-RM and Zeb1 were expressed in U2OS cells and then treated with 100 µg/ml cycloheximide (CHX) for time periods as indicated. Zeb1 protein expression was then assessed by Western blot. Loading was determined by β-actin. Relative pixel density of Zeb1 was normalized to respective loading control.

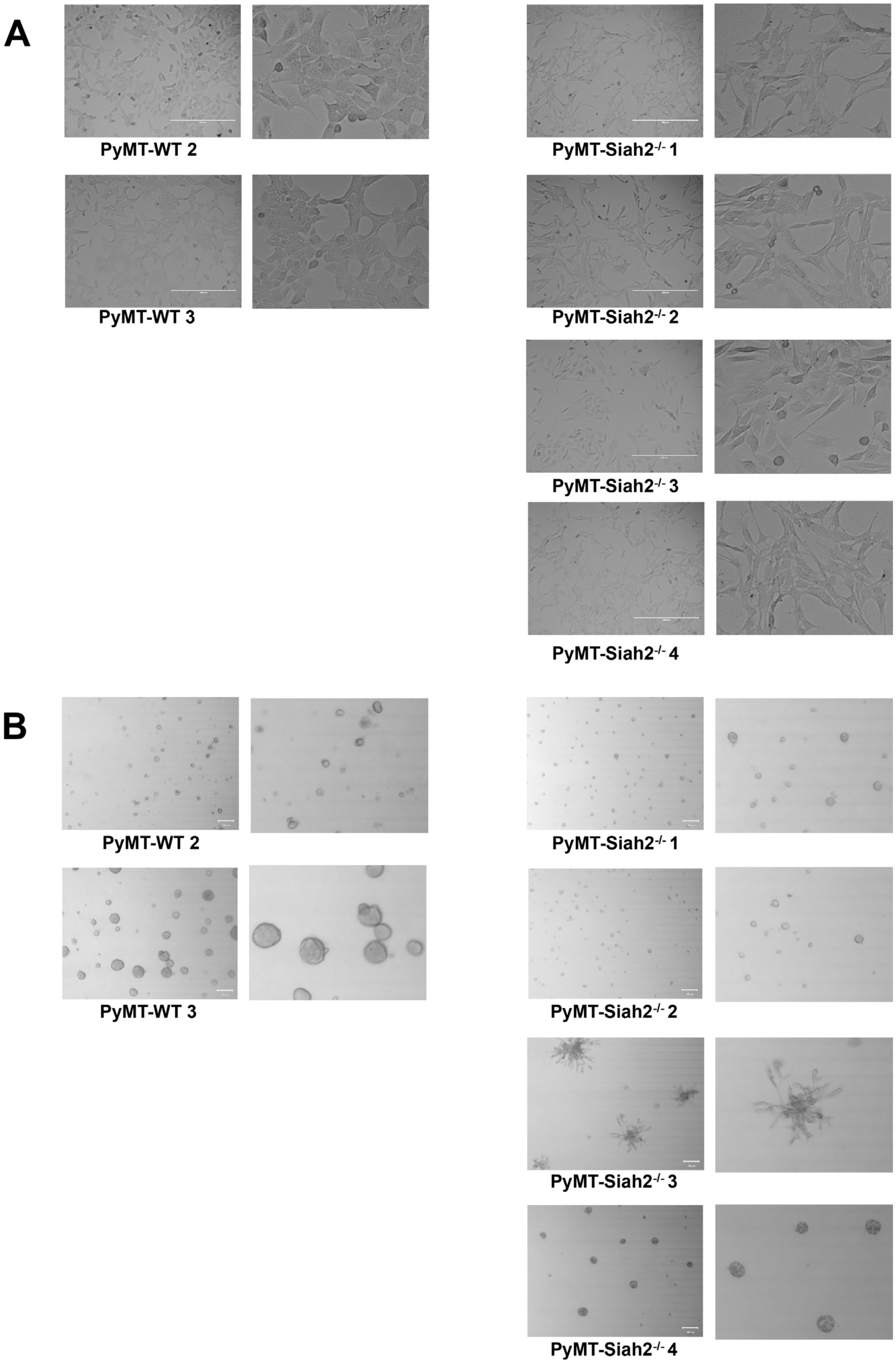

Supplementary Figure S4: PyMT-Siah2$^{-/-}$ cell lines undergo spontaneous EMT. (A) Phase contrast images for all PyMT-WT and PyMT-Siah2$^{-/-}$ cell lines not shown in Figure 3A. Scale bar represents 400 µm. **(B)** Images of 3D growth for all PyMT-WT and PyMT-Siah2$^{-/-}$ cell lines not shown in Figure 3B. Scale bar represents 100 µm.

(*Continued*)

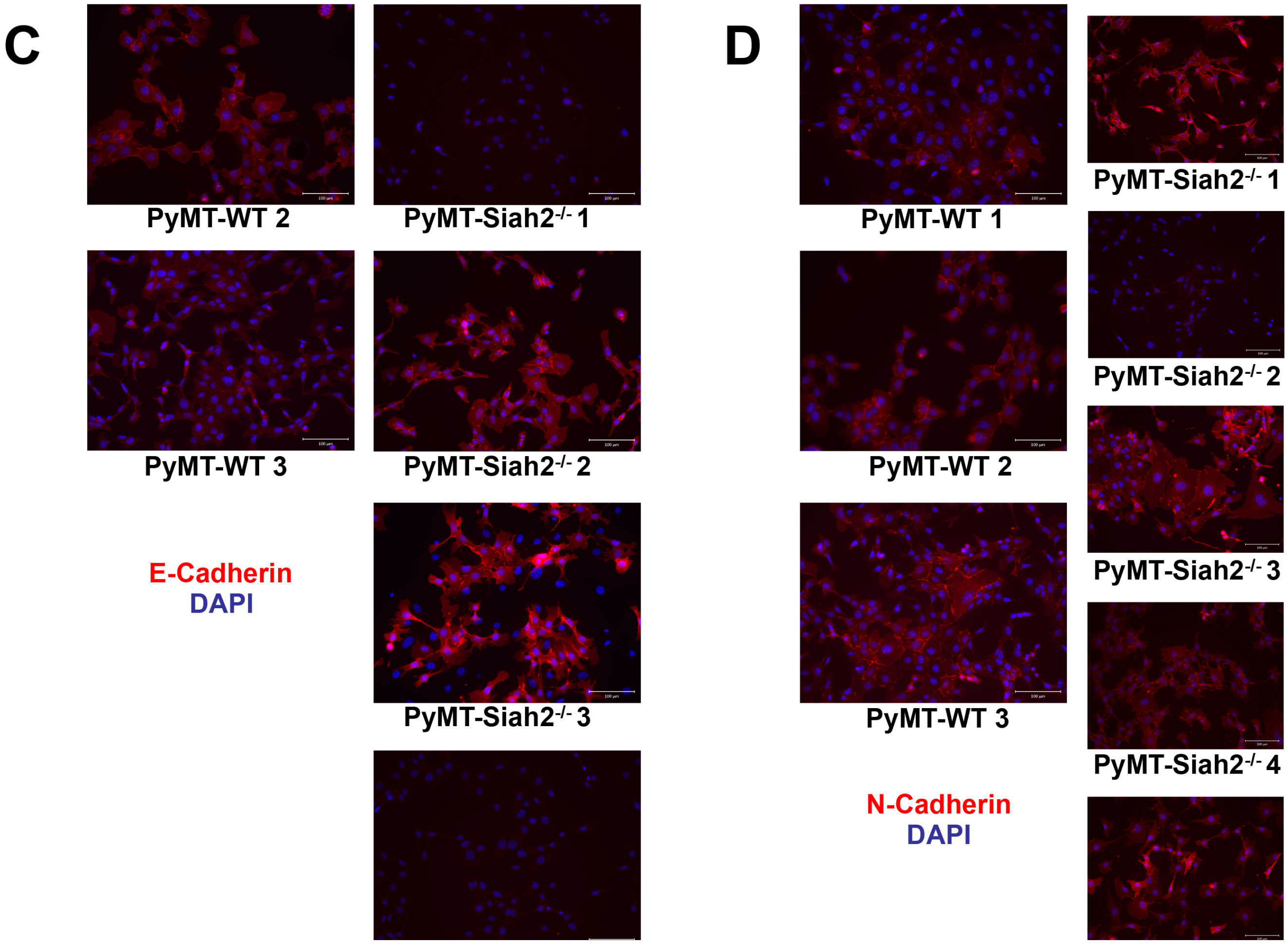

Supplementary Figure S4 (*Continued*): (C) E-cadherin immunofluorescence staining for all PyMT-WT and PyMT-Siah2$^{-/-}$ cell lines not displayed in Figure 4C. Scale bar represents 100 μm. **(D)** N-cadherin immunofluorescence staining for all PyMT-WT and PyMT-Siah2$^{-/-}$ cell lines. Scale bar represents 100 μm.

(*Continued*)

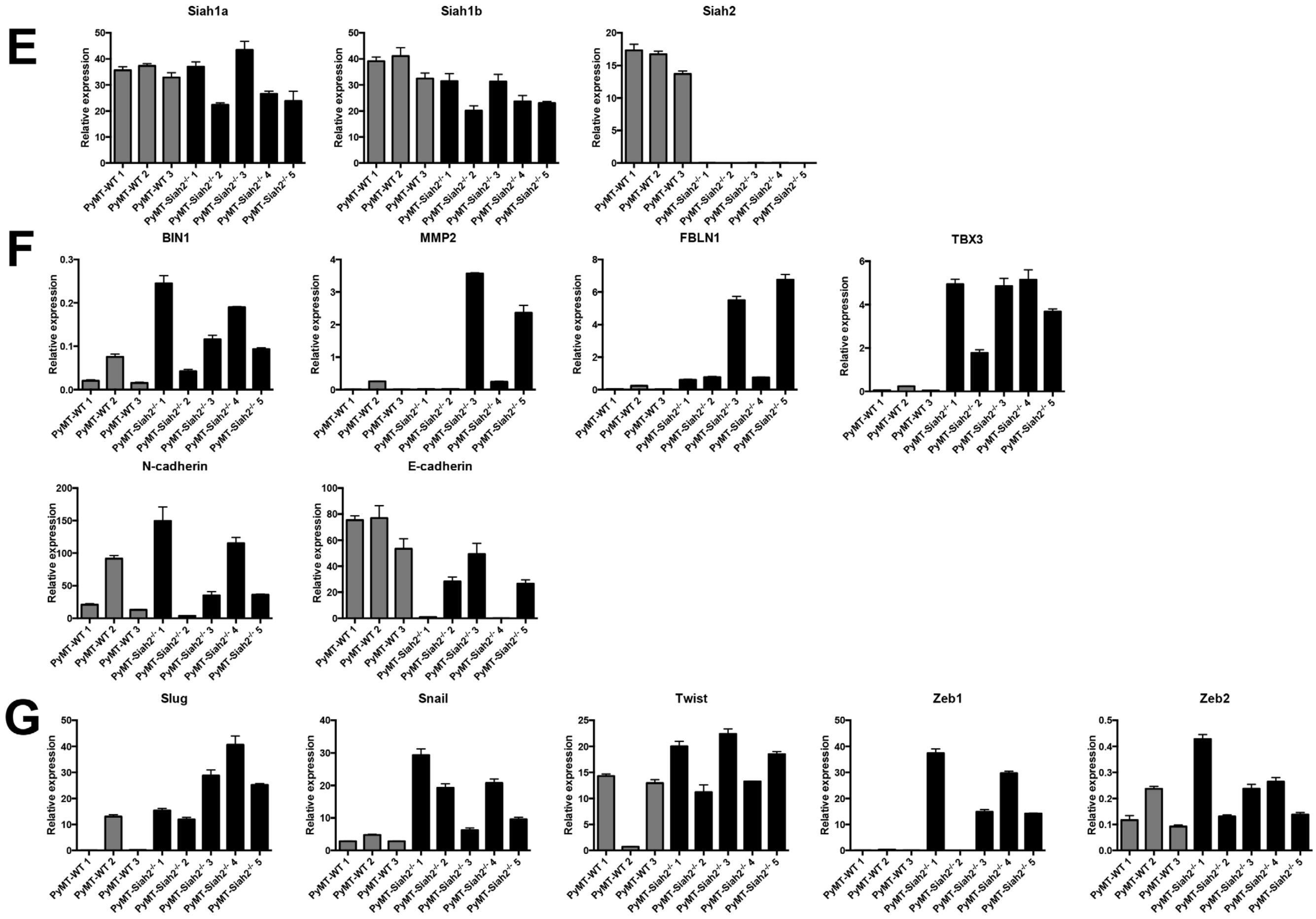

Supplementary Figure S4 (*Continued*): (E) Siah1a, Siah1b and Siah2 gene expression levels, as measured by qPCR, of PyMT-WT and PyMT-Siah2$^{-/-}$ cell lines. **(F)** mRNA expression of mesenchymal genes as determined by qPCR for each individual PyMT-WT and PyMT-Siah2$^{-/-}$ cell line. **(G)** mRNA expression levels of EMT transcription factors for each individual cell line. All qPCR experiments were performed in triplicate per technical repeat (three technical repeats). Data shown as mean ± SEM.
